# Genetic Variants Associated with Gestational Hypertriglyceridemia and Pancreatitis

**DOI:** 10.1371/journal.pone.0129488

**Published:** 2015-06-16

**Authors:** Sai-Li Xie, Tan-Zhou Chen, Xie-Lin Huang, Chao Chen, Rong Jin, Zhi-Ming Huang, Meng-Tao Zhou

**Affiliations:** 1 The First Affiliated Hospital of Wenzhou Medical University, Wenzhou, China; 2 Ren-Ji Study, Wenzhou Medical University, Wenzhou, China; Technische Universität München, GERMANY

## Abstract

Severe hypertriglyceridemia is a well-known cause of pancreatitis. Usually, there is a moderate increase in plasma triglyceride level during pregnancy. Additionally, certain pre-existing genetic traits may render a pregnant woman susceptible to development of severe hypertriglyceridemia and pancreatitis, especially in the third trimester. To elucidate the underlying mechanism of gestational hypertriglyceridemic pancreatitis, we undertook DNA mutation analysis of the lipoprotein lipase (*LPL*), apolipoprotein C2 (*APOC2*), apolipoprotein A5 (*APOA5*), lipase maturation factor 1 (*LMF1*), and glycosylphosphatidylinositol-anchored high-density lipoprotein-binding protein 1 (*GPIHBP1*) genes in five unrelated pregnant Chinese women with severe hypertriglyceridemia and pancreatitis. DNA sequencing showed that three out of five patients had the same homozygous variation, p.G185C, in *APOA5* gene. One patient had a compound heterozygous mutation, p.A98T and p.L279V, in *LPL* gene. Another patient had a compound heterozygous mutation, p.A98T & p.C14F in *LPL* and *GPIHBP1* gene, respectively. No mutations were seen in *APOC2* or *LMF1* genes. All patients were diagnosed with partial LPL deficiency in non-pregnant state. As revealed in our study, genetic variants appear to play an important role in the development of severe gestational hypertriglyceridemia, and, p.G185C mutation in *APOA5* gene appears to be the most common variant implicated in the Chinese population. Antenatal screening for mutations in susceptible women, combined with subsequent interventions may be invaluable in the prevention of potentially life threatening gestational hypertriglyceridemia-induced pancreatitis.

## Introduction

Hypertriglyceridemia-induced pancreatitis (HTP) is a rare yet serious complication of pregnancy, usually developing in the late gestation. It is a potentially life threatening condition, both for the mother and the fetus.[1] In normal pregnancy, there is a 2- to 4-fold increase in serum triglyceride (TG) levels due to increased hepatic synthesis of very-low-density lipoproteins (VLDL) in response to elevated estrogen levels and reduced lipoprotein lipase (LPL) activity.[2] However, this moderate physiological hypertriglyceridemia (HTG) is seldom associated with any adverse clinical consequences. However, severe HTG with a fasting plasma TG level of more than 1000 mg/dl (11.3 mmol/l), is known to be an independent risk factor for acute pancreatitis (AP).[3]

Women with a preexisting genetic abnormality in TG metabolism usually manifest modestly elevated TG level in non-pregnant state, which increases markedly during pregnancy.[4] Hypertriglyceridemia-induced pancreatitis in pregnant women is a well-known disorder. Earlier studies have largely been confined to the clinical features and management aspects, without much consideration of the genetic determinants.[5–7] The first genetic investigation of gestational HTP brought to light a homozygoous mutation in *LPL* gene responsible for impaired LPL activity.[8] To date, only a few individual cases have been reported wherein the genetic mutations of *LPL* and apoE polymorphism were implicated in the causation of gestational HTG and HTP.[9–12] However, in one such reported case, genetic variant could not be identified.[13]

LPL is a critical enzyme in TG metabolism, which hydrolyses TG-rich lipoprotein to free fatty acids, using apolipoprotein C2 (APOC2) as a cofactor. An impaired LPL activity results in massive accumulation of chylomicrons and fasting HTG.[14] In 2001, another modulator of LPL function, apolipoprotein A5 (APOA5) was reported.[15] Further, mutations in both *APOC2* and *APOA5* genes have been reported in patients with HTG.[16, 17] Recent studies have identified two new proteins involved in TG metabolism, namely, lipase maturation factor 1 (LMF1) and glycosylphosphatidylinositol-anchored high-density lipoprotein-binding protein 1 (GPIHBP1), making them the new candidate genes for the study of HTG.[18, 19]

Early identification of susceptible individuals with genetic defects, and appropriate management is a key strategy for reducing the risk of HTP during pregnancy. As part of the study aimed at elucidating the underlying mechanism of gestational HTP, we undertook DNA analysis of five pregnant women with a history of HTP, to identify any mutations in *LPL*, *APOC2*, *APOA5*, *LMF1*, and *GPIHBP1* genes.

## Materials and Methods

### Subjects

Our study was approved by the Ethics Committee of the First Affiliated Hospital of Wenzhou Medical University. Written informed consent was obtained from all study subjects.

Patient enrollment was conducted at the First Affiliated Hospital of Wenzhou Medical University, between June 2008 and January 2013. All patients had a history of developing HTP during pregnancy. The diagnosis of acute pancreatitis (AP) was made according to the Atlanta classification of acute pancreatitis (year 2012).[20] Fasting plasma TG concentration was greater than 1000 mg/dl (> 11.3 mmol/l) in all patients. Patients with a history of gallstones or secondary HTG (such as diabetes mellitus, hypothyroidism, nephritic syndrome, alcohol consumption, and usage of any medication known to affect lipid metabolism) were excluded from the study. A total of five unrelated women, all of Han Chinese descent, with gestational HTP were enrolled in the study. Clinical and laboratory findings of these five patients are presented in *Table 1*.

**Table 1:** Clinical and laboratory findings in the study population.

Patient	Age (years)	Obstetrical history	GA onset (weeks)	Genotype	TG (mmolL^-1^)	TC (mmolL^-1^)	Complications	Family history of HTG	TG level in follow-up time (mmolL^-1^)	Duration of follow-up (months)
1	28	G1P0	37	p.L279V/p.A98T	79.00	38.40	Pancreatic pseudocyst	Mother/HTP Two sisters/moderate HTG	3.2–4.5	26
2	22	G2P0	31	p.A98T /p.C14F	21.67	8.03	ARDS	Mother/mild HTG	1.9–2.8	21
3	30	G2P1	32	p.G185C	28.07	18.24	peritonitis	Nobody	2.0–4.4	37
4	31	G2P1	35	p.G185C	59.80	24.00	Pancreatic pseudocyst and peritonitis	Mother’s brother/HTP	1.6–5.2	13
5	27	G1P0	34	p.G185C	20.64	25.69	ARDS	Nobody	2.1–4.5	58

GA, gestational age; TC, total cholesterol; TG, total triglyceride; ARDS, acute respiratory distress syndrome.

Patient #1 was admitted with acute pancreatitis with a TG level of 79.0 mmol/l (normal- 1.7 mmol/l). An emergency cesarean section was conducted and a healthy baby girl was delivered. On day 4 postpartum, the patient’s serum TG level decreased to 15.4 mmol/l. At discharge, the reported TG level was 6.16 mmol/l.

Patient #2, had a history of miscarriage at 8 weeks of gestation, during her first pregnancy. During the second pregnancy, her serum TG level was found to be 21.67 mmol/l in the 31st week of gestation, at the time of admission for AP. The patient was discharged after 10 days of supportive treatment, with a TG level of 4.18 mmol/l. She was prescribed lipid-lowering diet and was under close medical supervision until 37 weeks of gestation, at which point she underwent elective cesarean section and delivered a healthy baby boy. Her pre-operative TG level was found to be 15.38 mmol/l.

**Fig 1:**
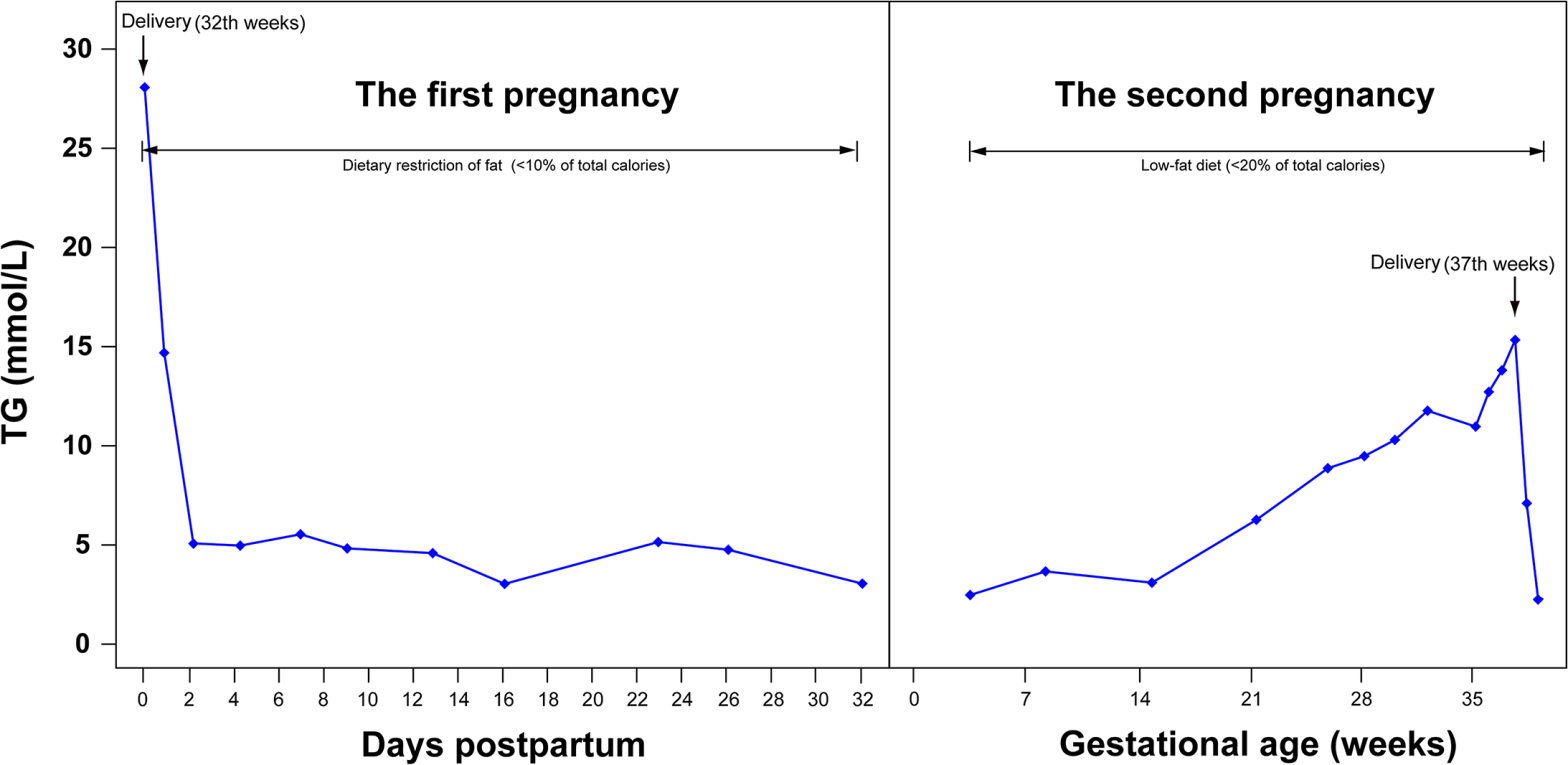
Plasma triglyceride levels and percent contribution of dietary fat in the total caloric intake are shown during two pregnancies and postpartum in Patient 3#.

First pregnancy of patient #3 terminated in fetal death at 32 weeks of gestation due to HTP. Serum TG level at that time was 28.07 mmol/l. Prior to and during her second pregnancy, she was on restricted dietary fat alongside a close monitoring of plasma lipids levels. An elective cesarean section was conducted at 37th week of gestation delivering a healthy girl child. The preoperative TG level was 16.13 mmol/l. Plasma TG levels during the immediate postpartum period after the first pregnancy and during the second pregnancy are presented in Fig 1.

Patient #4, first presented with epigastric pain at 38th week of gestation but did not develop AP. Her TG level was 15.9 mmol/l. The pregnancy ended with the delivery of a healthy baby girl. During her second pregnancy, she did not seek medical care until her admission at 35 weeks of gestation with AP and TG level of 24 mmol/l. Emergency cesarean section was performed and a healthy female baby delivered. She was discharged from hospital after being treated for AP. Her TG level at the time of discharge was 4.29 mmol/l.

Patient #5 was not diagnosed with severe HTG until she developed AP at 34 weeks of gestation. Her TG level was 20.64 mmol/l. Emergency caesarean section was performed and a healthy female baby delivered. Her plasma TG level fell from 14.38 mmol/l to 10.71 mmol/l in 24 hours post-delivery. Later, the patient was shifted to Intensive Care Unit for therapeutic plasma exchange (TPE). Her plasma TG level fell from 10.71 mmol/l to 5.68 mmol/l after one TPE.

None of the five patients have since developed AP.

Ten healthy adults, 5 men and 5 women, were enrolled in the study as controls. Their blood samples were drawn and tested for plasma LPL mass and activity.

### Measurement of plasma LPL mass and activity

Blood samples after overnight fasting were collected from cases and controls in Na-EDTA vials, 15 min after administration of intravenous heparin (60 IU/kg body weight). LPL enzyme activity was measured as per method described previously.[21] Enzyme activity was expressed as U/ml. LPL mass was measured using a sandwich-ELISA with a Human Lipoprotein Lipase ELISA Kit (Nanjing Jiancheng, Nanjing, China). The enzyme levels were expressed as ng of LPL per ml of plasma.

### Analysis of candidate genes

Blood was collected in 5 ml Na-EDTA vials; genomic DNA was extracted from 2 ml whole blood, as per the manufacturer’s instructions (Gentra Puregene Blood kit, QIAGEN, Germany). All coding regions and the intron-exon boundaries of *LPL*, *APOC2*, *APOA5*, *LMF1*, *GPIHBP1* genes were amplified and bidirectionally sequenced using the Sanger method, based on dideoxy chain-termination technology as has been earlier reported in a study.[22]

### Species examination

To explore the evolutionary conservation of the mutation, the protein sequences of APOA5 of select organisms were downloaded from NCBI (http://www.ncbi.nlm.nih.gov). Then, alignment of multiple sequences was performed, which indicates the evolutionary conservation, using ClustalX software.[23] The protein structure model of APOA5 was constructed by homology modeling using Swiss-model sever (http://swissmodel.expasy.org/).

## Results

### DNA analysis

DNA sequence analysis of the *LPL*, *APOC2*, *APOA5*, *LMF1*, *and GPIHBP1* genes revealed that patient #3, #4, and #5 had the same homozygosity of the p.G185C variant (c.553G>T, rs2075291) in the *APOA5* gene. The p.G185C variant is a G to T transition at the first nucleotide of codon 185, with a substitution of a cysteine for a glycine residue. No other DNA alteration was detected in all the exons and exon-intron boundaries of the *LPL*, *APOC2*,*GPIHBP1 or LMF1* genes. Genetic analysis of patient #1 demonstrated a compound heterozygote for mis-sense mutation, p.L279V and p.A98T in *LPL* gene. The mis-sense mutation p.L279V at exon 6 was a CTG→GTG change in codon 279 of the *LPL* gene, leading to L→V amino acid substitution in the LPL protein The mis-sense mutation p.A98T (c.292G>A, rs145657341) is a GCC→ACC change in codon 98 of the *LPL* gene, leading to an A→T amino acid substitution. On genetic analysis of patient #2, a compound heterozygosity for p.A98T and a known mis-sense mutation, p.C14F in *GPIHBP1* gene was found. The mis-sense mutation of p.C14F (c.41G>T, rs11538389) is a TGC→TTC change in codon 14 of the *GPIHBP1* gene, leading to a C→F amino acid substitution. There were no mutations found in *APOC2*, *APOA5 or LMF1* genes.

### Detection of post-heparin LPL activity and mass levels

**Fig 2:**
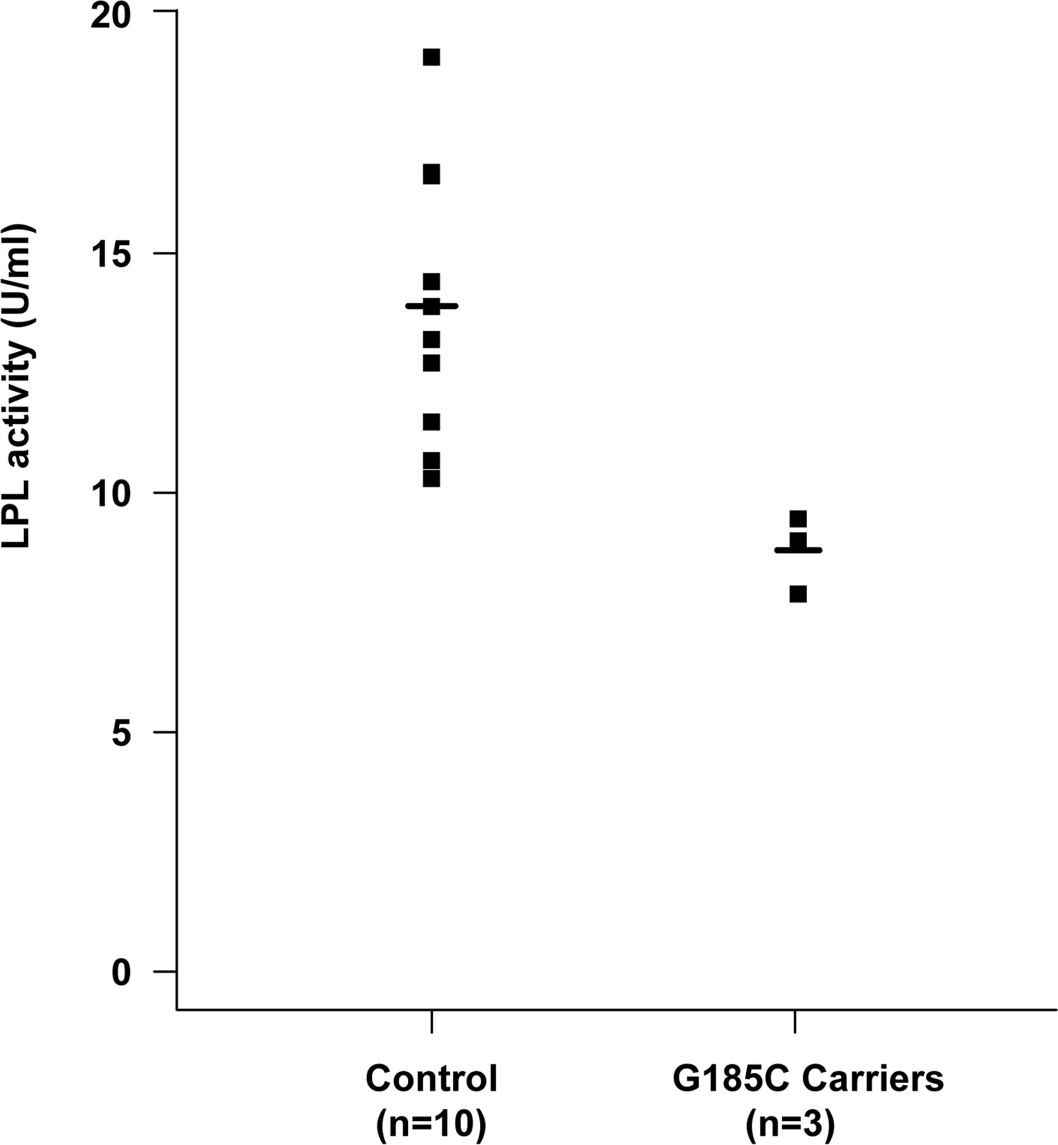
A Scatter plot of lipoprotein lipase activity levels in post-heparin plasma from carriers of the p.G185C mutation and controls.

We then detected post-heparin LPL activity and mass levels in patients during non-pregnant state and in normal controls. Compared to the mean for the control group, all the five patients had low LPL activity. Post-heparin plasma LPL activity levels in ten controls and three carriers of the p.G185C mutation are represented in Fig 2. Three p.G185C carriers had a group mean, which was 63.28% of the control mean. Carriers of p.L279V / p.A98T and p.A98T / p.C14F had LPL activity that was 65.1% and 42.18% lower than the control group, respectively. All of patients had partial LPL activity, while the LPL mass was close to normal.

### Species examination

**Fig 3:**
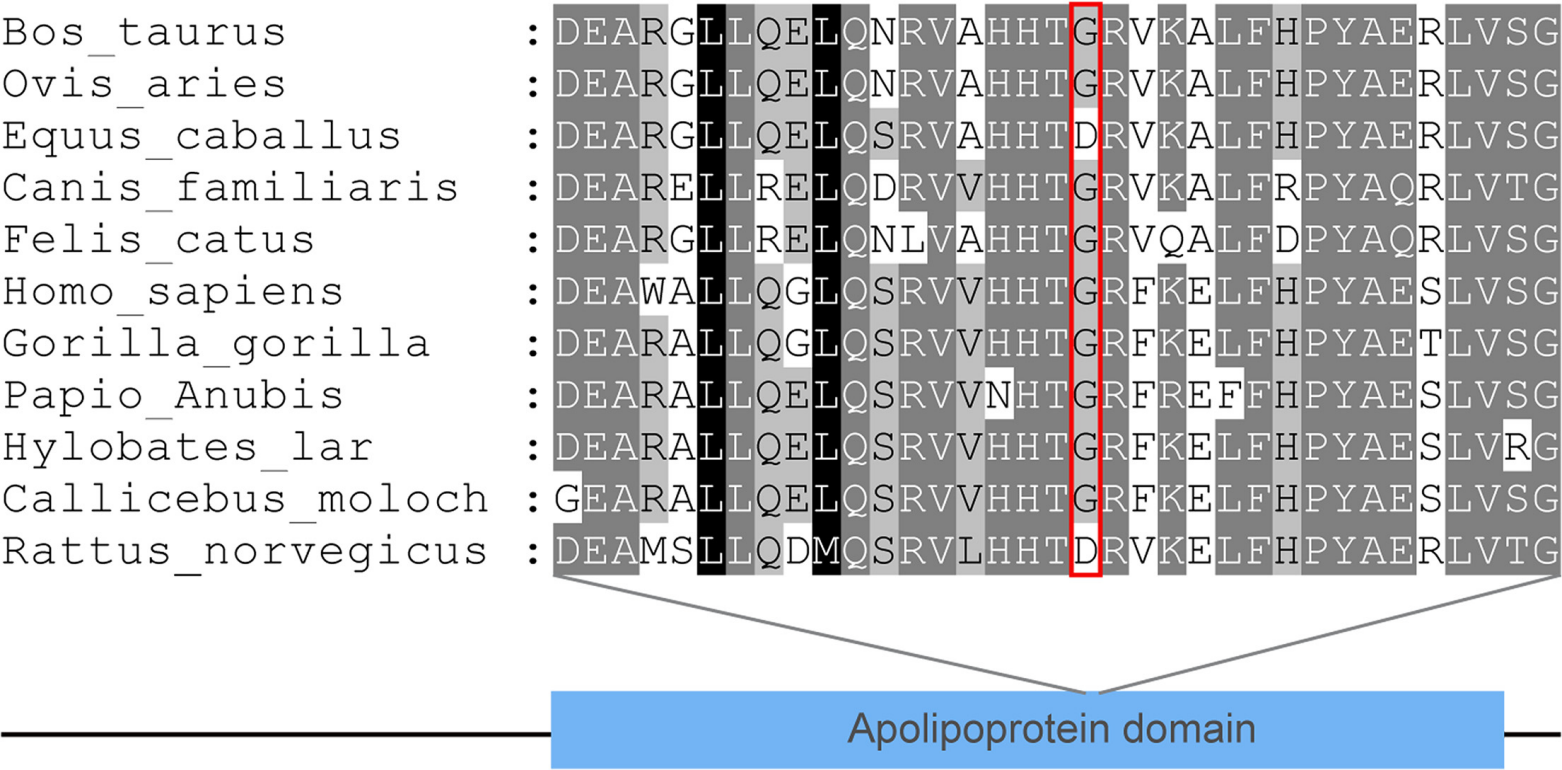
Evolutionary conservation of the G185 amino acid residue: G185 residue is relatively conserved across the species examined.

**Fig 4:**
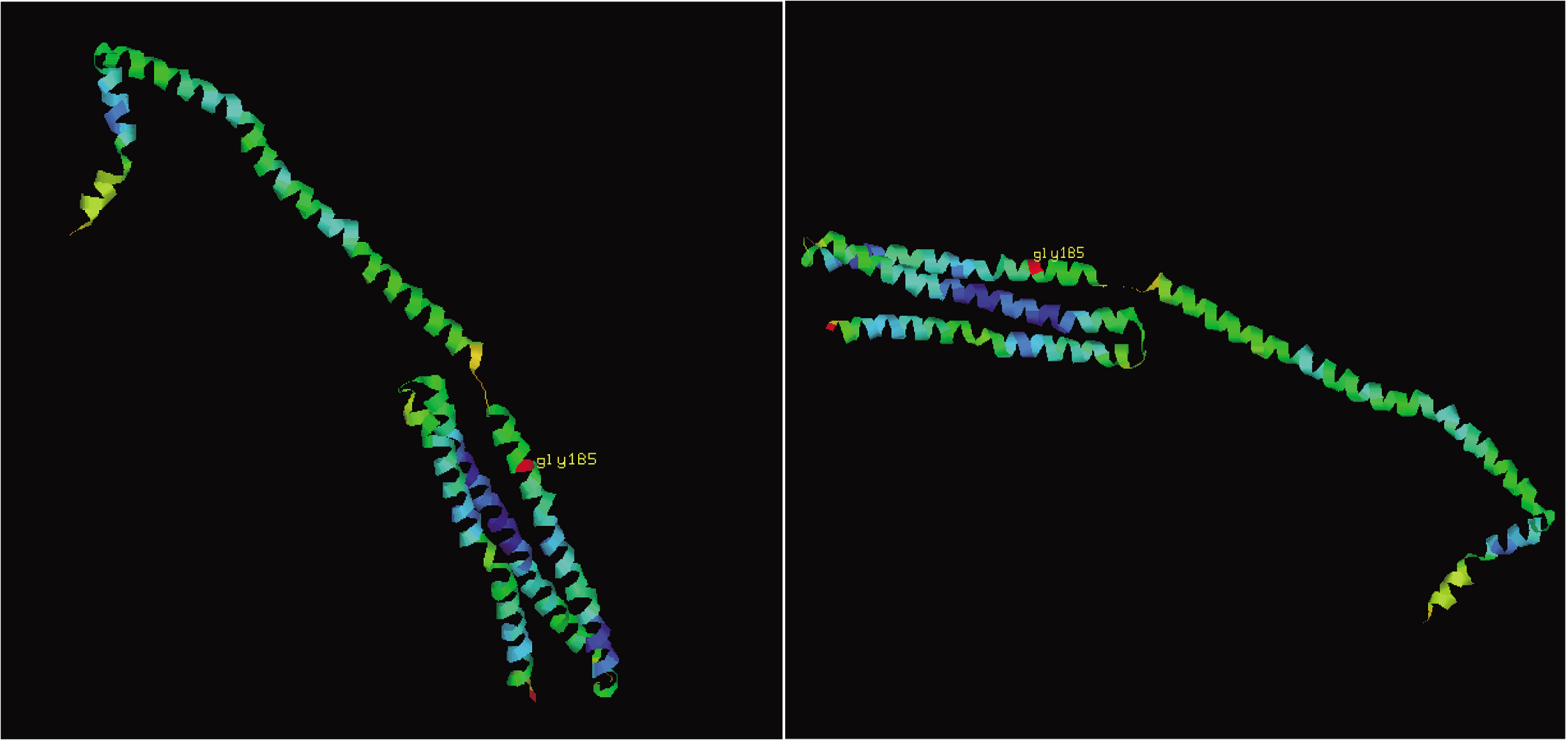
The protein structure model of APOA5 constructed by homology modeling.

We examined evolutionary conservation of the G185 amino acid residue across various species and we found that G185 residue is relatively conserved among different species (Fig 3). Additionally, we found that the p.G185C mutation leads to the amino acid change in the APOA1/C3/A4 domain, which contains several 22 residue repeats which form a pair of alpha helices (Fig 4).

## Discussion

Genetic variation in the *APOA5* locus, such as, p.S19W, p.G185C, p.V153M and -1131T>C, have been shown to be significantly associated with elevated plasma TG levels in human.[24–26] In particular, homozygous p.G185C mutation is strongly associated with extreme HTG.[27, 28]

The p.G185C variant of the APOA5 gene was first described in 2003.[26] Although several studies have reported p.G185C polymorphism of APOA5 gene in various populations, it has most commonly been found in Chinese people. T allele carriers have higher TG levels and different ethnic groups have different frequency of T allele. The frequency of T allele was reported to be absent in Caucasians,[29] while it is very low (0.004–0.006)[30, 31] in Turkish people. On the contrary, it was higher in Chinese (0.033–0.076)[26, 28, 32–35] and highest in Japanese (0.062–0.105).[36, 37] All subjects identified in these studies were homozygoous for p.G185C mutation and had pronounced hypertriglyceridemia. However, there was no reference to development of AP.

The precise mechanism of the effect of APOA5 on TG metabolism is still unclear. According to Fruchart-Najib, APOA5 may lower plasma TG levels by increasing LPL activity.[38] Merkel reported that APOA5 accelerates plasma hydrolysis of TG-rich lipoproteins by facilitating interaction with proteoglycan-bound LPL, and APOA5 acted as an allosteric LPL activator in the natural lipolytic system.[39] To further assess the disease-causing potential of p.G185C mutation, evolutionary conservation of the G185 amino acid residue was compared across different species. And the result demonstrated the G185 was relatively conserved, suggesting that this residue may play a critical role in the function of LPL. Furthermore, all the p.G185C carriers in our study had partial LPL activity in non-pregnant state. The p.G185C mutation probably affects the proteoglycan-bond of LPL, thereby reducing the LPL activity.

Patients with severe HTG usually show Fredrickson type 1 hyperlipidemia, fasting serum lactescent with clinical features of recurrent attacks of pancreatitis, hepatosplenomegaly, lipemia retinalis and eruptive xanthomas. HTG is usually categorized as primary and secondary. Excessive alcohol consumption, diabetes mellitus, nephritic syndrome and use of certain drugs are common causes of secondary HTG.[40] Primary severe HTG is usually attributed to genetic defects, which may be further aggravated by physiological factors such as pregnancy.

Mutations in the *LPL* gene are common factors in the pathogenesis of HTP during pregnancy.[9–11] In this study, one patient was a compound heterozygote for mis-sense mutation p.A98T and p.L279V in *LPL* gene, and another was compound heterozygote for p.A98T in *LPL gene*, and had a known mis-sense mutation, p.C14F, in *GPIHBP1* gene. Both of these patients had low LPL activity but almost normal mass. Both variants of p.A98T and p.C14F had been described in individuals with familial LPL deficiency and HTG.[41, 42]

The general therapy for gestational HTP is to alleviate TG levels during the acute episodes with a very low-fat diet (< 10% of total caloric intake), hospitalization for intravenous fluid therapy and parenteral nutrition. Specific therapies like continuous intravenous heparin and TPE also have been described in various case reports[43, 44] Heparin can dramatically decrease plasma TG levels, followed by gradual rebound due to increased hepatic degradation.[45] The effects of TPE are also significant but transient in the absence of concomitant fat intake restriction. Both heparin and TPE can only serve as a short-term treatment option and their utility and safety have not been thoroughly assessed.

Home diet restriction and hospital admission for intensive dietary control was not found to be sufficient for preventing progression to AP, and early cesarean section may still be required, as was done in patient #2 and #3. Though there is no clear evidence to suggest that delivery could be essential for rapid improvement of TG levels, our patients showed a dramatic decrease of TG levels and positive outcomes after delivery.[7, 8]

Our findings indicate that genetic variants play an important role in severe hypertriglyceridemia during pregnancy, and, p.G185C mutation in *APOA5* gene might be a common variant in individuals of Chinese descent. However, further studies with larger sample size are required to validate these findings. Early diagnosis and intervention are key factors for successful outcome. Dietary restriction of fat and close monitoring of lipid levels during pregnancy is of utmost importance, while, cesarean section may be an appropriate treatment choice to prevent the development of AP and its associated complications.

## References

[1] De Chalain TM, Michell WL, Berger GM. Hyperlipidemia, pregnancy and pancreatitis. Surgery, gynecology & obstetrics. 1988;167(6):469–73. . 3187871

[2] Alvarez JJ, Montelongo A, Iglesias A, Lasuncion MA, Herrera E. Longitudinal study on lipoprotein profile, high density lipoprotein subclass, and postheparin lipases during gestation in women. Journal of lipid research. 1996;37(2):299–308. . 9026528

[3] Yadav D, Pitchumoni CS. Issues in hyperlipidemic pancreatitis. Journal of clinical gastroenterology. 2003;36(1):54–62. . 12488710 10.1097/00004836-200301000-00016

[4] Suga S, Tamasawa N, Kinpara I, Murakami H, Kasai N, Onuma T, et al Identification of homozygous lipoprotein lipase gene mutation in a woman with recurrent aggravation of hypertriglyceridaemia induced by pregnancy. Journal of internal medicine. 1998;243(4):317–21. . 9627147 10.1046/j.1365-2796.1998.00306.x

[5] Crisan LS, Steidl ET, Rivera-Alsina ME. Acute hyperlipidemic pancreatitis in pregnancy. American journal of obstetrics and gynecology. 2008;198(5):e57–9. 10.1016/j.ajog.2008.01.003 . 18359475

[6] Shenhav S, Gemer O, Schneider R, Harats D, Segal S. Severe hyperlipidemia-associated pregnancy: prevention in subsequent pregnancy by diet. Acta obstetricia et gynecologica Scandinavica. 2002;81(8):788–90. . 12174168 10.1034/j.1600-0412.2002.810819.x

[7] Hsia SH, Connelly PW, Hegele RA. Successful outcome in severe pregnancy-associated hyperlipemia: a case report and literature review. The American journal of the medical sciences. 1995;309(4):213–8. . 7900743 10.1097/00000441-199504000-00005

[8] Ma Y, Liu MS, Ginzinger D, Frohlich J, Brunzell JD, Hayden MR. Gene-environment interaction in the conversion of a mild-to-severe phenotype in a patient homozygous for a Ser172—>Cys mutation in the lipoprotein lipase gene. The Journal of clinical investigation. 1993;91(5):1953–8. 10.1172/JCI116414 8486765 PMC288190

[9] Ma Y, Ooi TC, Liu MS, Zhang H, McPherson R, Edwards AL, et al High frequency of mutations in the human lipoprotein lipase gene in pregnancy-induced chylomicronemia: possible association with apolipoprotein E2 isoform. Journal of lipid research. 1994;35(6):1066–75. . 8077845

[10] Murugasu CG, Armstrong G, Creedon G, Cavanna JS, Galton DJ, Tomkin GH. Acute hypertriglyceridaemic pancreatitis in a pregnant Indian: a new lipoprotein lipase gene mutation. Journal of the Royal Society of Medicine. 1998;91(4):205–7. 9659309 10.1177/014107689809100410 PMC1296643

[11] Henderson H, Leisegang F, Hassan F, Hayden M, Marais D. A novel Glu421Lys substitution in the lipoprotein lipase gene in pregnancy-induced hypertriglyceridemic pancreatitis. Clinica chimica acta; international journal of clinical chemistry. 1998;269(1):1–12. . 9498099 10.1016/s0009-8981(97)00144-7

[12] McGladdery SH, Frohlich JJ. Lipoprotein lipase and apoE polymorphisms: relationship to hypertriglyceridemia during pregnancy. Journal of lipid research. 2001;42(11):1905–12. . 11714860

[13] Eskandar O, Eckford S, Roberts TL. Severe, gestational, non-familial, non-genetic hypertriglyceridemia. The journal of obstetrics and gynaecology research. 2007;33(2):186–9. 10.1111/j.1447-0756.2007.00506.x . 17441893

[14] Fisher RM, Humphries SE, Talmud PJ. Common variation in the lipoprotein lipase gene: effects on plasma lipids and risk of atherosclerosis. Atherosclerosis. 1997;135(2):145–59. . 9430364 10.1016/s0021-9150(97)00199-8

[15] Pennacchio LA, Olivier M, Hubacek JA, Cohen JC, Cox DR, Fruchart JC, et al An apolipoprotein influencing triglycerides in humans and mice revealed by comparative sequencing. Science. 2001;294(5540):169–73. 10.1126/science.1064852 . 11588264

[16] Pennacchio LA, Rubin EM. Apolipoprotein A5, a newly identified gene that affects plasma triglyceride levels in humans and mice. Arteriosclerosis, thrombosis, and vascular biology. 2003;23(4):529–34. 10.1161/01.ATV.0000054194.78240.45 . 12615678

[17] Fojo SS, Brewer HB. Hypertriglyceridaemia due to genetic defects in lipoprotein lipase and apolipoprotein C-II. Journal of internal medicine. 1992;231(6):669–77. . 1619390 10.1111/j.1365-2796.1992.tb01256.x

[18] Peterfy M, Ben-Zeev O, Mao HZ, Weissglas-Volkov D, Aouizerat BE, Pullinger CR, et al Mutations in LMF1 cause combined lipase deficiency and severe hypertriglyceridemia. Nature genetics. 2007;39(12):1483–7. 10.1038/ng.2007.24 . 17994020

[19] Olivecrona G, Ehrenborg E, Semb H, Makoveichuk E, Lindberg A, Hayden MR, et al Mutation of conserved cysteines in the Ly6 domain of GPIHBP1 in familial chylomicronemia. Journal of lipid research. 2010;51(6):1535–45. 10.1194/jlr.M002717 20026666 PMC3035517

[20] Banks PA, Bollen TL, Dervenis C, Gooszen HG, Johnson CD, Sarr MG, et al Classification of acute pancreatitis—2012: revision of the Atlanta classification and definitions by international consensus. Gut. 2013;62(1):102–11. 10.1136/gutjnl-2012-302779 . 23100216

[21] Misra KB, Kim KC, Cho S, Low MG, Bensadoun A. Purification and characterization of adipocyte heparan sulfate proteoglycans with affinity for lipoprotein lipase. The Journal of biological chemistry. 1994;269(38):23838–44. . 8089154

[22] Surendran RP, Visser ME, Heemelaar S, Wang J, Peter J, Defesche JC, et al Mutations in LPL, APOC2, APOA5, GPIHBP1 and LMF1 in patients with severe hypertriglyceridaemia. Journal of internal medicine. 2012;272(2):185–96. 10.1111/j.1365-2796.2012.02516.x 22239554 PMC3940136

[23] Thompson JD, Gibson TJ, Plewniak F, Jeanmougin F, Higgins DG. The CLUSTAL_X windows interface: flexible strategies for multiple sequence alignment aided by quality analysis tools. Nucleic acids research. 1997;25(24):4876–82. 9396791 10.1093/nar/25.24.4876 PMC147148

[24] Pennacchio LA, Olivier M, Hubacek JA, Krauss RM, Rubin EM, Cohen JC. Two independent apolipoprotein A5 haplotypes influence human plasma triglyceride levels. Human molecular genetics. 2002;11(24):3031–8. . 12417524 10.1093/hmg/11.24.3031

[25] Talmud PJ, Hawe E, Martin S, Olivier M, Miller GJ, Rubin EM, et al Relative contribution of variation within the APOC3/A4/A5 gene cluster in determining plasma triglycerides. Human molecular genetics. 2002;11(24):3039–46. . 12417525 10.1093/hmg/11.24.3039

[26] Kao JT, Wen HC, Chien KL, Hsu HC, Lin SW. A novel genetic variant in the apolipoprotein A5 gene is associated with hypertriglyceridemia. Human molecular genetics. 2003;12(19):2533–9. 10.1093/hmg/ddg255 . 12915450

[27] Pullinger CR, Aouizerat BE, Movsesyan I, Durlach V, Sijbrands EJ, Nakajima K, et al An apolipoprotein A-V gene SNP is associated with marked hypertriglyceridemia among Asian-American patients. Journal of lipid research. 2008;49(8):1846–54. 10.1194/jlr.P800011-JLR200 18441017 PMC2444008

[28] Lee MJ, Chien KL, Chen MF, Stephenson DA, Su TC. Overweight modulates APOE and APOA5 alleles on the risk of severe hypertriglyceridemia. Clinica chimica acta; international journal of clinical chemistry. 2013;416:31–5. 10.1016/j.cca.2012.10.054 . 23178747

[29] Hubacek JA, Adamkova V, Ceska R, Poledne R, Horinek A, Vrablik M. New variants in the apolipoprotein AV gene in individuals with extreme triglyceride levels. Physiological research / Academia Scientiarum Bohemoslovaca. 2004;53(2):225–8. . 15046561

[30] Can Demirdogen B, Sahin E, Turkanoglu Ozcelik A, Bek S, Demirkaya S, Adali O. Apolipoprotein A5 polymorphisms in Turkish population: association with serum lipid profile and risk of ischemic stroke. Molecular biology reports. 2012;39(12):10459–68. 10.1007/s11033-012-1926-z . 23065249

[31] Hodoglugil U, Tanyolac S, Williamson DW, Huang Y, Mahley RW. Apolipoprotein A-V: a potential modulator of plasma triglyceride levels in Turks. Journal of lipid research. 2006;47(1):144–53. 10.1194/jlr.M500343-JLR200 . 16258166

[32] Yin RX, Li YY, Liu WY, Zhang L, Wu JZ. Interactions of the apolipoprotein A5 gene polymorphisms and alcohol consumption on serum lipid levels. PloS one. 2011;6(3):e17954 10.1371/journal.pone.0017954 21423763 PMC3056790

[33] Tang Y, Sun P, Guo D, Ferro A, Ji Y, Chen Q, et al A genetic variant c.553G > T in the apolipoprotein A5 gene is associated with an increased risk of coronary artery disease and altered triglyceride levels in a Chinese population. Atherosclerosis. 2006;185(2):433–7. 10.1016/j.atherosclerosis.2005.06.026 . 16046221

[34] Zhai G, Wen P, Guo L, Chen L. Association of APOA5 c.553G>T polymorphism with type 2 diabetes mellitus in a Chinese population. Clinical chemistry and laboratory medicine: CCLM / FESCC. 2006;44(11):1313–6. 10.1515/CCLM.2006.255 . 17087641

[35] Hsu LA, Ko YL, Chang CJ, Hu CF, Wu S, Teng MS, et al Genetic variations of apolipoprotein A5 gene is associated with the risk of coronary artery disease among Chinese in Taiwan. Atherosclerosis. 2006;185(1):143–9. 10.1016/j.atherosclerosis.2005.05.031 . 16054149

[36] Matsunaga A, Arishima H, Niimura H, Zhang B, Uehara Y, Ohwaki K, et al Strong linkage disequilibrium and association of -1131T>C and c.553G>T polymorphisms of the apolipoprotein A5 gene with hypertriglyceridemia in a Japanese population. Circulation journal: official journal of the Japanese Circulation Society. 2007;71(5):746–52. . 17457003 10.1253/circj.71.746

[37] Hishida A, Morita E, Naito M, Okada R, Wakai K, Matsuo K, et al Associations of apolipoprotein A5 (APOA5), glucokinase (GCK) and glucokinase regulatory protein (GCKR) polymorphisms and lifestyle factors with the risk of dyslipidemia and dysglycemia in Japanese—a cross-sectional data from the J-MICC Study. Endocrine journal. 2012;59(7):589–99. . 22517333 10.1507/endocrj.ej11-0310

[38] Fruchart-Najib J, Bauge E, Niculescu LS, Pham T, Thomas B, Rommens C, et al Mechanism of triglyceride lowering in mice expressing human apolipoprotein A5. Biochemical and biophysical research communications. 2004;319(2):397–404. 10.1016/j.bbrc.2004.05.003 . 15178420

[39] Merkel M, Loeffler B, Kluger M, Fabig N, Geppert G, Pennacchio LA, et al Apolipoprotein AV accelerates plasma hydrolysis of triglyceride-rich lipoproteins by interaction with proteoglycan-bound lipoprotein lipase. The Journal of biological chemistry. 2005;280(22):21553–60. 10.1074/jbc.M411412200 . 15774484

[40] Tsuang W, Navaneethan U, Ruiz L, Palascak JB, Gelrud A. Hypertriglyceridemic pancreatitis: presentation and management. The American journal of gastroenterology. 2009;104(4):984–91. 10.1038/ajg.2009.27 . 19293788

[41] Merkel M, Eckel RH, Goldberg IJ. Lipoprotein lipase: genetics, lipid uptake, and regulation. Journal of lipid research. 2002;43(12):1997–2006. . 12454259 10.1194/jlr.r200015-jlr200

[42] Yamamoto H, Onishi M, Miyamoto N, Oki R, Ueda H, Ishigami M, et al Novel combined GPIHBP1 mutations in a patient with hypertriglyceridemia associated with CAD. Journal of atherosclerosis and thrombosis. 2013;20(10):777–84. . 23831619 10.5551/jat.18861

[43] Sivakumaran P, Tabak SW, Gregory K, Pepkowitz SH, Klapper EB. Management of familial hypertriglyceridemia during pregnancy with plasma exchange. Journal of clinical apheresis. 2009;24(1):42–6. 10.1002/jca.20192 . 19160449

[44] Swoboda K, Derfler K, Koppensteiner R, Langer M, Pamberger P, Brehm R, et al Extracorporeal lipid elimination for treatment of gestational hyperlipidemic pancreatitis. Gastroenterology. 1993;104(5):1527–31. . 8482465 10.1016/0016-5085(93)90366-k

[45] Nasstrom B, Olivecrona G, Olivecrona T, Stegmayr BG. Lipoprotein lipase during continuous heparin infusion: tissue stores become partially depleted. The Journal of laboratory and clinical medicine. 2001;138(3):206–13. 10.1067/mlc.2001.117666 . 11528374

